# Dexamethasone induced Dectin-1 activation enhances NLRP3 inflammasome activation

**DOI:** 10.3389/fimmu.2025.1656288

**Published:** 2025-09-12

**Authors:** Ruth-Miriam Koerber, Sebastian Oberbeck, Philipp Kotthoff, Solveig N. Daecke, Peter Brossart, Stefanie A. E. Held

**Affiliations:** ^1^ Department of Medicine III, University Hospital Bonn, Bonn, Germany; ^2^ Mildred Scheel School of Oncology, University Hospital Bonn, Medical Faculty, Bonn, Germany

**Keywords:** dectin-1, NLRP3 inflammasome, tolerogenic dendritic cells, fungal infections, immunological regulation

## Abstract

Systemic candidiasis is a serious complication in immunocompromised patients, with Candida albicans emerging as the most common opportunistic pathogen. In various therapeutic treatment regimens the immunosuppressive agent Dexamethasone is used. Dexamethasone itself impairs the function of dendritic cells and reduces thereby their capacity for T-cell proliferation through the activation of Dectin-1 by β-glucans. In the present study, we reveal that these tolerogenic dendritic cells (Dex-DCs) have an increased secretion of IL-1ß and IL-18 when stimulated with β-glucans. We show an increased formation of ASC specks, which are crucial for recruiting pro-caspase-1, indicating an elevated inflammasomal activity. In line with this, we were able to show that treatment of tolerogenic dendritic cells with a NLRP3 inhibitor prior to Dectin-1 stimulation normalized the secretion of IL-1ß and IL-18. Furthermore, the addition of Caspase- and Syk-inhibitors led to diminished inflammasome activation as well as to less pyroptosis and apoptosis in response to β -glucan stimulation. Finally, we identified elevated production of reactive oxygen species (ROS) upon β-glucan stimulation in DexDCs as a possible mechanism for apoptosis induction as it can be reversed by the treatment with a specific anti-Dectin-1 antibody. Moreover, the underlying mechanism of the NLRP3 activation seems to be mediated through mitochondrial DNA release induced by mitochondrial ROS. Taken together, the present study demonstrates that Dectin-1 stimulation of tolerogenic DCs can result in severe pro-inflammatory responses due to cytokine release and subsequent NLRP3 inflammasome activation. In conclusion, the application of NLRP3 inflammasome inhibitors to patients treated with corticosteroids like Dexamethasone may significantly improve their outcome as they might be well-protected against local or severe systemic fungal infections.

## Graphical Abstract

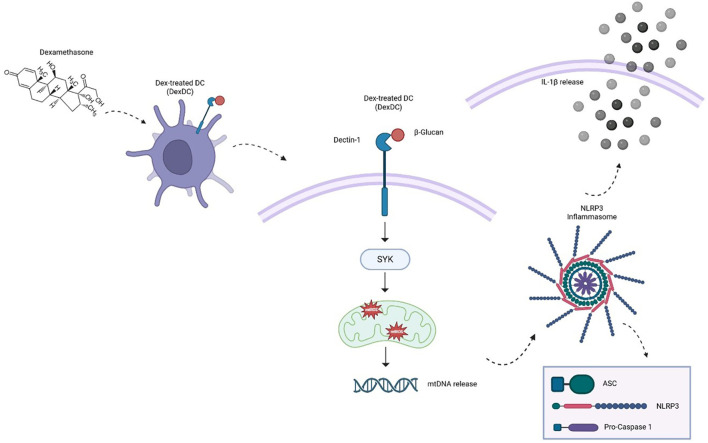

## Introduction

Systemic candidiasis, a serious complication in patients with compromised immune systems, is a significant cause of mortality. Among the various Candida species, Candida albicans (C. albicans) has emerged as a prevalent opportunistic pathogen (1). The widely used pharmaceutical agent dexamethasone has been demonstrated to induce immunosuppression, which is attributed to a suppression of differentiation, function and proliferation of T-cells. This is revealed by downregulation of co-stimulatory molecules such as CD28 and an impaired cytokine production (e.g. IL-6, TNF-alpha). Furthermore, dexamethasone decreases antigen presentation by dendritic cells, which limits again T-cell activation and induction of ROS can further activate pro-apoptotic pathways like NF-kappaB. (2). Treatment of monocyte-derived dendritic cells (moDC) with dexamethasone has been shown to result in a deficiency of co-stimulatory molecules and an impaired capacity for effective T cell proliferation. Additionally, these cells exhibit an augmented release of IL-10 and an increased induction of regulatory T cells (Tregs) (3).

A critical component of fungal cell walls, including components of candida yeast, are ß-glucans. These can be sensed via Dectin-1, a major receptor on DCs, macrophages, and neutrophils (4) and is a member of the group of pattern-recognition receptors (PRRs) (5). For example, Curdlan, a bacterial ß- (1, 3)-glucan polysaccharide, is a specific ligand of Dectin-1, capable of triggering innate immune responses (6). It has been demonstrated that human DC can activate spleen tyrosine kinase (Syk) via Dectin-1 in a TLR2-dependent manner, subsequently inducing NF-kappaB (NFκB)-associated inflammatory responses. However, it is imperative to note that while β-glucan-associated stimulation of Dectin-1 can orchestrate a variety of immune stimulatory effects of innate and adaptive immune responses, an adequate DC function is essential to sufficiently control fungal infections (7). Kotthoff et al. demonstrated that Dex-DC are impaired in their immunological function as relators of fungal β-glucans with Dectin-1 (6).

The distinct T helper cell population of TH17 cells plays a crucial role in effective anti-candida immunity, and their proliferation is promoted by, for example, IL-1ß (8). Consequently, it is postulated that both pro-inflammatory cytokines IL-1ß and IL-18 play a substantial role in anti-fungal immune defenses via T cell-mediated IL-17 and INF-γ production (9, 10). The primary source for maturation of cytosolic IL-1ß and IL-18 are inflammasomes. The most thoroughly studied inflammasome is the NOD-, LRR- and pyrin domain-containing protein 3 (NLRP3) (11). The subsequent process of NLRP3 association with the adaptor protein ASC (apoptosis speck-like protein containing a caspase recruitment domain) leads to the cleavage of caspase-1. ASC specks are micrometer-sized protein structures formed by the inflammasome adaptor ASC. The formation of ASC specks is a hallmark of inflammasome activation (12), which in turn activates the procession of pro-IL-1ß and pro-IL-18 (13). This series of events results in pyroptotic cell death. Apart from inflammasome-mediated IL-1ß maturation, non-canonical caspase-8 inflammasome and dectin-1-dependent IL-1ß activating mechanisms have been described (14). Since ß-glucans are highly immunostimulatory, further research is necessary to better understand the associated sensing and effector pathways, especially in situations involving immune compromise with Dexamethasone.

Crucial players of our immune system are so called dendritic cells (DCs), whose main task is to recognize and present antigens to other immune cells. Two main groups can be distinguished: immature and tolerogenic DCs. Tolerogenic DCs can be discriminated phenotypically from immature DCs, for example, by the expression of CD80, CD83, and CD86. Functionally, tolerogenic DCs are involved in mediating immune tolerance. Our research group has demonstrated this in previous studies (15). In the present study, we observed a significant secretion of the cytokines IL-1ß and IL-18 in tolerogenic dendritic cells (Dex-DC) compared to immature dendritic cells (iDC) when stimulated with Curdlan, indicating enhanced inflammasome activation. Additionally, Dex-DC showed an increased formation of ASC specks, which are crucial for recruiting pro-caspase-1, suggesting amplified inflammasome activity in these cells.

Actually, there is a tolerogenic–inflammatory paradox existing in Dex-DCs: Dex-DCs are usually programmed to be immunosuppressive and tolerogenic. Nevertheless, tolerogenic DCs can robustly release pro-inflammatory signals when stimulated via the Dectin-1 pathway, inducing a strong innate inflammatory response (16). This paradox arises because Dex-DCs exhibit suppression of adaptive immunity but can still activate innate inflammatory responses under certain stimuli. As mentioned above, Dex-DCs appear to have a tolerogenic phenotype (diminished expression of costimulatory molecules like CD80 and CD83, upregulation of anti-inflammatory mediators like MERTK, IL-10) with a reduced ability to activate T cells. These features promote expansion of regulatory T-cells (Tregs) and suppress adaptive immune responses, particularly antigen-specific T-cell activation (17). On the other hand, Dectin-1 stimulation triggers Syk-dependent and Syk-independent signaling, activating NF-κB and leading to production of pro-inflammatory cytokines (IL-1β, TNF-α, IL-6, etc.) and inflammasome activation. We observed, that Dectin-1 is sufficient to trigger innate pro-inflammatory responses in previously tolerogenic Dex-DCs, overriding their immunosuppressive programming at the level of innate immunity. More precise, Dectin-1 stimulation leads to the activation of cytokine secretion (IL-1β, IL-18), characteristic of robust innate immunity. Taken together, tolerogenic programming targeted by dexamethasone is most effective on the adaptive arm [T-cell regulation (17)], while innate pathways [e.g., Dectin-1, TLR (18)] can remain responsive to specific danger signals, enabling a paradoxical inflammatory response even by cells considered tolerogenic. In this investigation, we sought to elucidate a potential mechanistic pathway in response to β-glucan-stimulated human tolerogenic dendritic cells (DCs) treated with dexamethasone (Dex-DC). Our findings revealed that the activation of Dectin-1 by β-glucans can induce pyroptosis and apoptosis in tolerogenic DCs. In the presence of dexamethasone, Dectin-1-mediated activation of mDCs has been shown to result in increased secretion of IL-1ß, leading to enhanced inflammation. This process may play a role in the development of local and systemic pathologic effects associated with fungal infections.

## Methods

### Ethics statement

This study was conducted in accordance with the Declaration of Helsinki and approved by the Institutional Ethics Committee of the University of Bonn, North-Rhine Westphalia, Germany (grant number 173/09). Buffy coats for human monocyte isolation were obtained from healthy, voluntary blood donors of the University Hospital Bonn. Written informed consent was obtained from all volunteers for blood donation and further processing of blood samples for scientific purposes by the blood bank/transfusion medicine of the University Hospital of Bonn. All experiments were performed in accordance with relevant guidelines and regulations.

### Media and reagents

Cells were cultured in RPMI 1640 containing glutamax-I, supplemented with 10% inactivated fetal calf serum (RP10 medium) and 1% penicillin/streptomycin (Invitrogen, California, USA). All reagents not otherwise indicated were purchased from Sigma-Aldrich. Curdlan was purchased from Wako Chemicals USA, Inc. (Virginia, USA). Zymosan A was purchased from InvivoGen (San Diego, USA). Oridonin was purchased from Cayman Chemical (Michigan, USA). R406 was purchased from Cayman Chemical (Michigan, USA). zVAD (Z-VAL-ALA-DL-Asp-fluoromethylketone) was purchased from Bachem (Switzerland).

### Generation of iDCs and Dex-DCs

Human monocyte-derived DCs (iDCs) were generated from peripheral blood by plastic adherence as described previously (19, 20). Adherent monocytes were cultured in RP10 medium supplemented with GM-CSF (100 ng/mL; Leukine, Liquid Sargramostim) and IL-4 (20 ng/ml; R&D Systems). Cytokines were added to the cells every other day.

To generate Dex-DC adherent monocytes were treated as described for iDC. Dexamethasone was added further at 100 nM every other day. In each case, equal amounts of EtOH were added as a control.

### Immunostaining for flow cytometry

iDC and Dex-DC were harvested, washed and stained using commercially available mAbs from Biolegend (ASC, clone HASC-71). Cells were, as indicated, stimulated for 18 h with Curdlan (at 100 µg/ml) prior to flow cytometric analysis. For determination of ASC-Specks, a Caspase-1 inhibitor (50 µM Belnacasan, Selleckchem) was added prior to Curdlan stimulation. Cells were then further processed for subsequent analysis.

Intracellular staining was carried out using the FoxP3/Transcription Factor Staining Buffer Set (Thermo Fisher Scientific). For exclusion of dead cells a ZOMBIE dye (Biolegend) was used. Data were acquired on a FACS Canto II (BD) and analyzed with FlowJo 10.8.1. (BD). The gating is shown examplarily in **Supplementary Figure S2**.

### Determination of cytokine production

Concentration of cytokines in cell-culture supernatants was determined using DuoSet^®^ ELISA Development Systems (R&D systems) according to the manufacturer’s instructions for IL-1ß and IL-18. Cells were harvested, washed and seeded in 24-well plates in fresh RP10 media prior to stimulation. Dectin-1 stimulation was accomplished by stimulation for 18 h with curdlan (both at 100 µg/ml) prior to cytokine measurement. Inhibitors were added to the cells 30 min prior to stimulation at the indicated concentrations.

### Detection of superoxide-anion generation

Dex-DC and iDC were washed and plated in 96-well cell-culture plate at 5 × 10^5^ per well in fresh RP10 medium and were allowed to settle for 12–24 h, after the aforementioned culturing conditions. DCs were incubated with Inhibitors at the indicated concentrations, MnTBAP (Sigma-Aldrich, California, USA), Oridonin or an anti-hDectin-1 antibody (Human Dectin-1 CLEC7A Antibody, Biotechne, Minnesoa, USA) 30 min prior to stimulation with the beta-glucan zymosan (100 µg/ml). At timepoint of stimulation the superoxide detection reagent (Invitrogen™, H2DCFDA) was applied to the DCs. Cell-permeable 2’,7’-dichlorodihydrofluorescein diacetate (H2DCFDA) is a chemically reduced form of fluorescein that is used as an indicator of reactive oxygen species (ROS) in living cells. Oxidation with any reactive oxygen species leads to the formation of a fluorescent product, 2’,7’-dichlorofluorescein, which we measured using flow cytometry. DCs were stimulated for 45 min with H2DCFDA before harvested and analysed.

### Statistical analysis

All experiments were performed at least two or three times, with representative experiments shown. Statistical analysis was performed with GraphPad 9 (Prism). The statistical tests used are indicated in the figure legends.

## Results

### Stimulation of DCs with the dectin-1 ligand curdlan leads to inflammasome activation and is enhanced in tolerogenic DCs.

The activation of Dectin-1 by β-glucans has been demonstrated to elicit immune system activation, accompanied by pro- and anti-inflammatory responses. The expression of HLA-DR- or PD-L1/2-positive dendritic cells and the measurement of cytokines have been identified as hallmarks of these responses. Our group has previously demonstrated that tolerogenic DCs (generated in the presence of dexamethasone) exhibit elevated levels of Dectin-1 expression (6).

**Figure 1:**
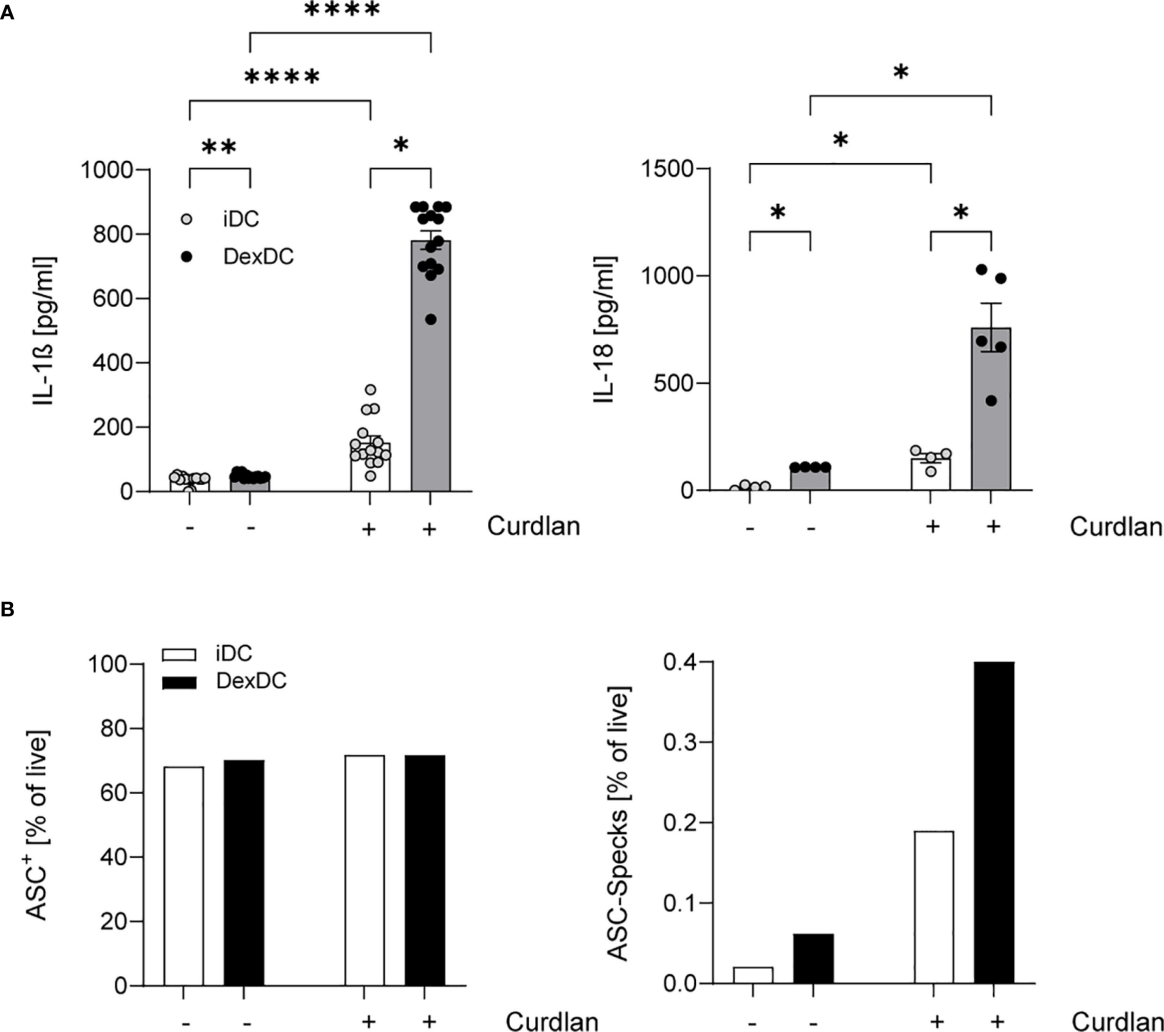
Dex-DC reveal a signature of inflammasome activation. **(A)** Supernatants of iDC and Dex-DC with and without Dectin-activation via Curdlan-stimulation were analyzed for IL-1ß and IL-18 secretion as described. Scatter bar plots show mean ± SEM with each dot representing the median of duplicates of four independent experiments. Kruskal-Wallis test was applied for statistical analysis. *P < 0.01, **P < 0.005, ****P < 0.0001. **(B)** Intracellular ASC expression (left) and specking ASC-molecules (right) in iDC and Dex-DC were measured by flow cytometry under unstimulated conditions or following curdlan stimulation. Percentage of positive cells/specks is indicated and one representative experiment of four is shown.

We analyzed the cytokine secretion of IL-1ß and IL-18 in supernatants of two distinct cell populations: tolerogenic DCs (Dex-DC) and immature DCs (iDC). These cells were stimulated with Curdlan (100 µg/ml), a known specific ligand for Dectin-1, with minimal TLR-stimulating properties. Our findings revealed a pronounced IL-1ß and IL-18 response in Dex-DC (**Figure 1A**). Given the established role of IL-1ß and IL-18 as the key effector cytokines of the NLRP3-inflammasome (21), we hypothesize that there is enhanced inflammasome activation in tolerogenic DCs. Conversely, we found elevated levels of the anti-inflammatory cytokine IL-10 in Dex-DC compared to iDC (**Supplementary Figure S1**).

A major event in NLRP3 inflammasome activation is the formation of so-called ASC-specks, which recruit pro-caspase-1 to its activating complex (22). Our observations reveal an augmented tendency for ASC Speck formation in Dex-DC in comparison to iDC, while the expression levels of non-specking ASC molecules remain comparable between Dex-DC and iDC (**Figure 1B**). These findings collectively suggest an amplified inflammasome activation within the context of tolerogenic DCs.

### Inflammasome activation in dectin-1 stimulated Dex-DC with curdlan can be abrogated by NLRP3-inhibition.

**Figure 2:**
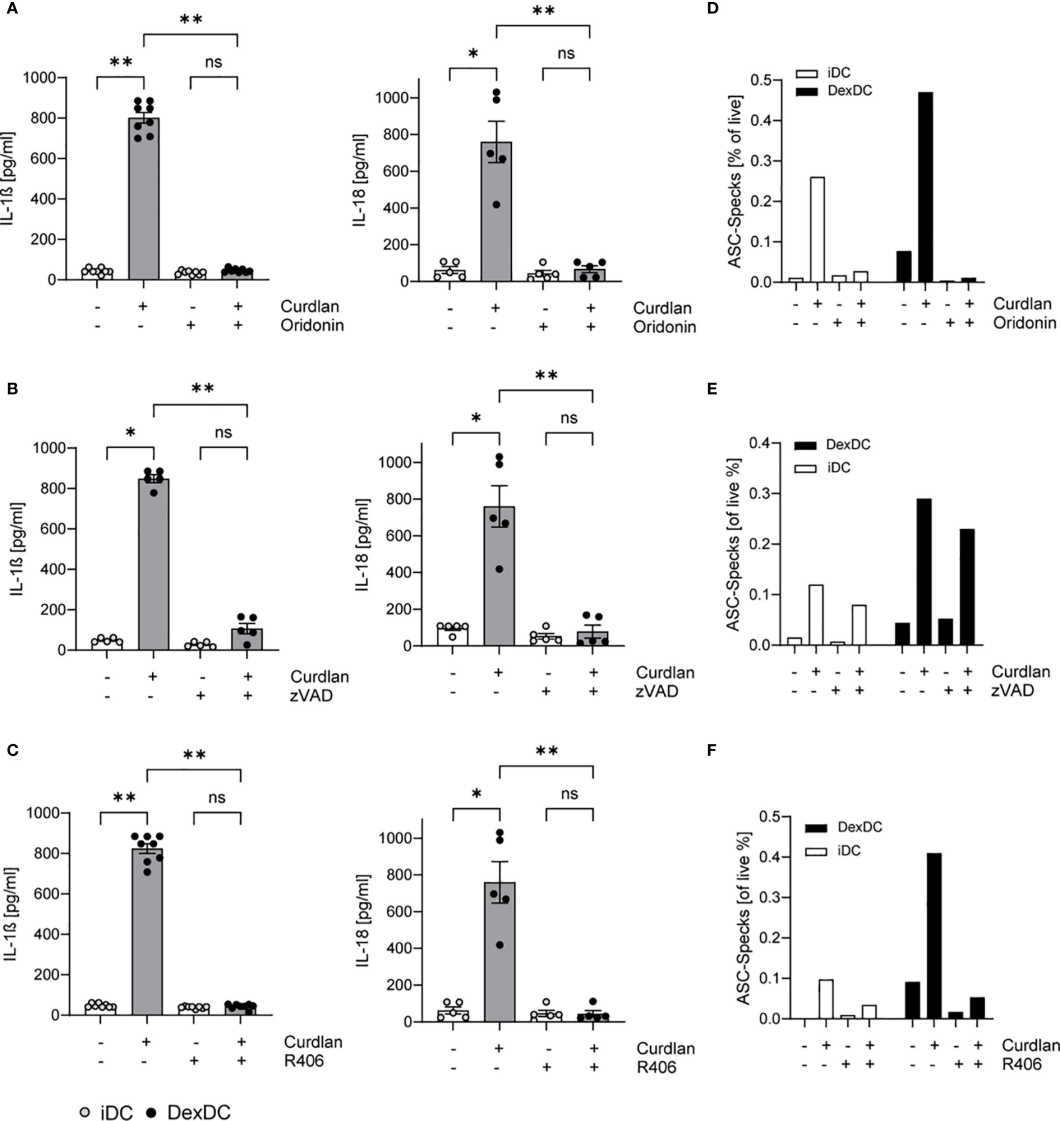
Activated Dectin-1 signaling in Dex-DC induces pyroptosis and apoptosis. iDC and Dex-DC were either unstimulated or stimulated with Curdlan and treated with a NLRP3-inhibitor (Oridonin), a pan-Caspase inhibitor (zVAD) and a Syk-inhibitor (R406) as indicated. **(A–C)** Supernatants of these cultures were analyzed for IL-1ß and IL-18 secretion as described in the methods section. Scatter bar plots show mean ± SEM with each dot representing the median of duplicates of independent experiments. Kruskal-Wallis test was applied for statistical analysis. *P < 0.01, **P < 0.005. **(D–F)** Specking ASC-molecules were measured by flow cytometry in iDCs and DEX-DC under unstimulated conditions or following curdlan stimulation. Percentage of specks is indicated and one representative experiment of four is shown.

To verify our hypothesis of NLRP3 induction in Dex-DC, we tested the specific NLRP3 inhibitor Oridonin (10 µM). The cells were treated with the inhibitor for 1.5 hours prior to Dectin-1 induction. The secretion of IL-1ß and IL-18 in the culture media of Dex-DC was observed, resulting in the normalization of cytokine levels to that of those of non-curdlan activated controls. A similar response was observed in iDCs (**Figure 2A**). The activation of the NLRP3 inflammasome typically results in pyroptosis. To assess whether apoptosis is also diminished in iDC and DexDC following Dectin-1 stimulation, we examined the effects of the pan-caspase inhibitor zVAD (10 µM). Following a 1.5-hour period of inhibitor treatment and stimulation with Curdlan (100 µg/ml), we measured IL-1ß and IL-18 in supernatants of iDCs and Dex-DCs. The analysis revealed a reduction in these cytokines after inhibitor treatment (**Figure 2B**). Furthermore, targeting the Dectin-1/Syk-pathway with R406, a known Syk-inhibitor (10 µM), in Dex-DCs also leads to reduced levels of IL-1ß and IL-18 (**Figure 2C**). Concomitant with the cytokine levels, a decrease in ASC-Specking cells was observed after inhibitor treatment in Dex-DC compared to iDC (**Figures 2D–F**).

Collectively, these observations demonstrate that Dex-DCs trigger both pyroptosis and apoptosis following Curdlan stimulation. Furthermore, the inhibition of Syk-signaling has been observed to result in a dampened inflammasome activation.

### Inflammasome activation in tolerogenic Dex-DC is ROS-dependent as a potential mechanism of NLRP3-activation.

**Figure 3:**
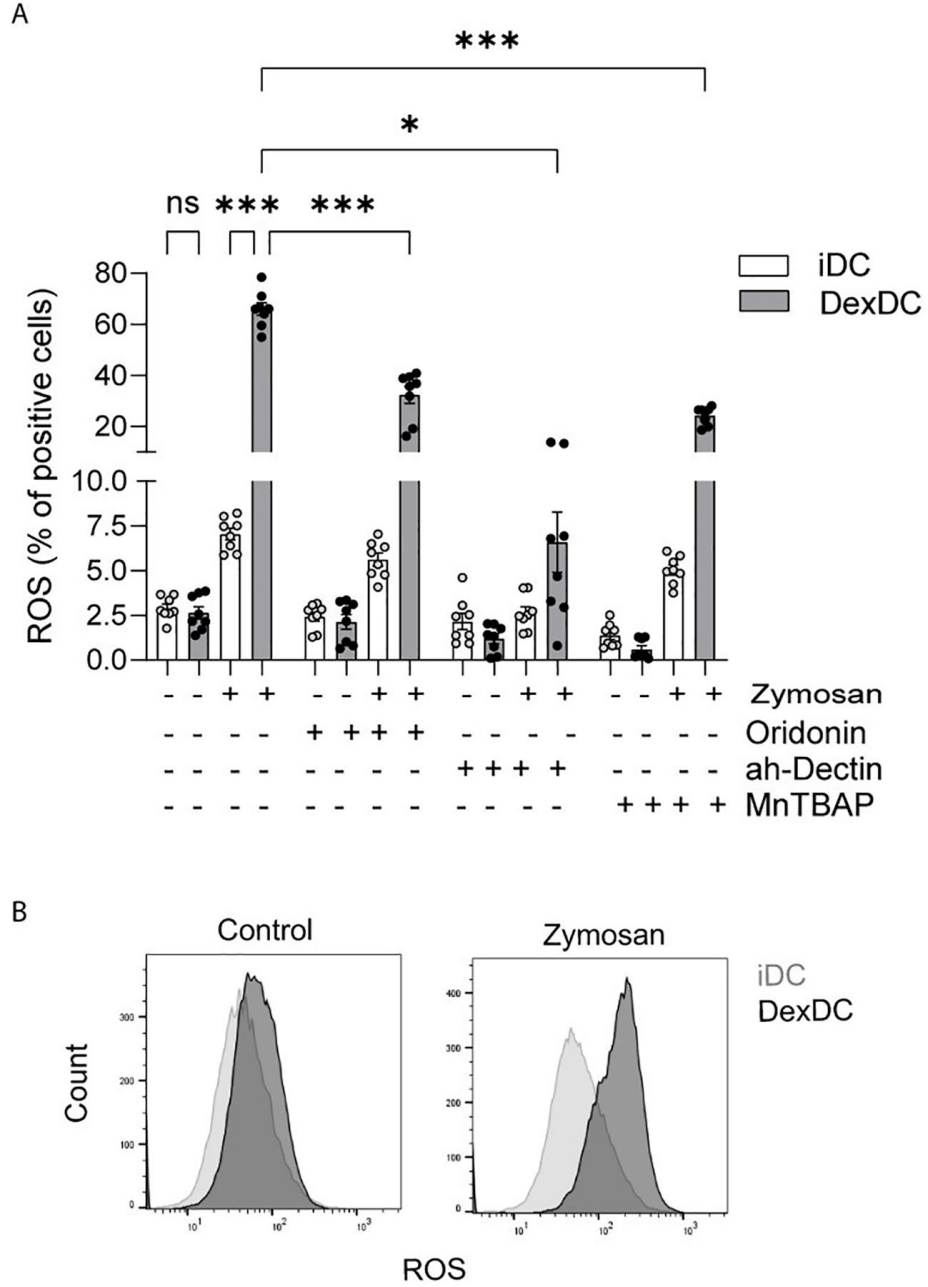
Dectin-1 activation leads to inflammasome activation via ROS-production. **(A)** iDC and Dex-DC were stimulated with Zymosan A, control solvent and anti-Dectin Antibody and MnTBAP as indicated. Superoxide-Anion production was measured by flow cytometry and percentages of ROS-positive cells are shown. Scatter bar plots show mean ± SEM with each dot representing a measurement result of four experiments in duplicates. Mann-Whitney-U-test was applied for statistical analysis. *P < 0.01, ***P < 0.001. **(B)** Staining histograms display gating of ROS-distribution of the afore mentioned experiment.

A variety of mechanisms have been identified that facilitate NLRP3 activation. The production of Reactive Oxygen Species (ROS) is a common activating event upstream of NLRP3 (23). In the present study, upon stimulation with the alternative β-glucan Zymosan A (100 µg/ml), a heightened level of ROS production was observed in Dex-DC in comparison to iDC. To validate signaling via Dectin-1, we treated both iDC and Dex-DC with a specific anti-Dectin-1 antibody (5 µg/ml) and found ROS-levels equivalent to control conditions, indicating a Dectin-1-mediated ROS release after fungal infections. Furthermore, anti-Dectin 1 treatment resulted in a protection from dexamethasone induced ROS production (**Figure 3A**).

The overproduction of ROS, also known as oxidative stress, has been demonstrated to result in cellular damage or dysfunction and can induce cell death. The production of ROS occurs in various cellular locations, including the mitochondria, the endoplasmic reticulum, and the peroxisomes (24). These ROS can either directly or indirectly activate the NLRP3 inflammasome. A potential direct pathway for NLRP3 activation involves the release of mitochondrial DNA (mtDNA), which is dependent on mitochondrial ROS (mtROS) (25). The release of mtDNA directly binds and activates NLRP3. To test this possible source of NLRP3 activating mtROS, we treated iDC and Dex-DC with the superoxide dismutase (SOD)-mimetic MnTBAP (0,4 mM). MnTBAP has been shown to neutralize superoxides in both extra- and intracellular compartments without scavenging nitric oxide (NO). In the context of Zymosan A-stimulated Dex-DC, the ability of MnTBAP to impede mtROS production was observed, although this effect was less pronounced in comparison to its effect on iDC (**Figures 3A, B**). These findings demonstrate a NO-independent ROS production in Dex-DC as a potential NLRP3-activating mechanism.

## Discussion

The utilization of immunosuppressive medications, such as dexamethasone, remains an indispensable component in the management of hematologic diseases and autoinflammatory processes. However, these treatments are frequently accompanied by fungal infections, particularly those caused by Candida species, with C. albicans being the most prevalent pathogen. Under normal conditions, colonization with C. albicans does not typically result in severe systemic infections. This is due to the protective effects of TH1 and TH17 cells against mucosal invasions, as well as the ability of neutrophils, monocytes/macrophages to prevent systemic candidiasis (26). The primary receptor responsible for detecting the Candida species via their fungal cell wall component of β-glucans is Dectin-1 (27). The well-studied C-type lectin receptor (CLR) Dectin-1 is predominantly expressed by dendritic cells (DCs), monocytes, and macrophages, leading to the production of a Syk-mediated pro-inflammatory response (28). Other mechanisms of action include signaling via TLR2 and TLR4 (29) and the formation of neutrophil extracellular traps (NETs) (30).

In this study, we demonstrate that dexamethasone-treated monocyte-derived dendritic cells (Dex-DC) elicit a robust pro-inflammatory response that is mediated by the NLRP3 inflammasome. The stimulation of Dectin-1 in Dex-DC resulted in the detection of a robust and characteristic NLRP3-mediated pro-inflammatory response, which was significantly more pronounced compared to iDC. The analysis revealed elevated levels of IL-1ß and IL-18, the principal effector cytokines of the NLRP3 inflammasome. The formation of ASC specks is a consequence of NLRP3 oligomerization, which is a prerequisite for the activation of this inflammasome (22). This process results in the cleavage of the effector-Caspase-1. Active Caspase-1 plays a pivotal role in the maturation of the cytokines into biologically active forms of pro-IL-1ß and pro-IL-18. In the present study, we observed a greater prevalence of activated, ASC-specking Dex-DC compared to iDC following Dectin-1 stimulation with Curdlan. Furthermore, the use of the specific NLRP3-inhibitor Oridonin leads to a downregulation of IL-1ß and IL-18 in DexDC and decreased counts of ASC specking DexDC. It has been established that NLRP3 plays a pivotal role in C. albicans-induced inflammation. This assertion is supported by the findings of Vonk et al., who demonstrated that IL-1ß deficient mice exhibited an elevated mortality rate due to an inadequate TH1 response following C. albicans infection (31). Additionally, it is postulated that the activation of the NLRP3 inflammasome is pivotal in differentiating between the colonization and invasion of Candida species (32). Our findings indicate that Dex-DC responds to Curdlan stimulation with augmented inflammasome activation, underscoring the heightened risk of systemic fungal infections associated with the administration of immunosuppressive corticosteroids. Our findings indicate that the administration of Oridonin, a NLRP3 inhibitor, or R406, a Syk inhibitor, results in the attenuation of inflammasome activation. Cheng et al. observed in their experiments that the Dectin-1/Syk-pathway is apparently only in part responsible for the IL-1ß response in fungal infections (33). A priming step for inflammasome activation is typically required. In the present experiments, an IL-1ß and IL-18 response was observed in Dex-DC directly after Curdlan stimulation, without the application of other prior agents. Consequently, these findings underscore the notion that NLRP3 induction can occur in the absence of a priming step, thereby emphasizing the direct inflammasome activation via Dectin-1-signaling. Given that Dex-DC has been observed to exaggerate IL-1ß and IL-18 levels in comparison to iDC, it can be deduced that potential IL-1ß associated effector functions of non-pyroptotic cells may be impaired in Dex-DC.

A physiological response to fungal infections of host cells and the fungus itself is the creation of ROS, leading to oxidative stress (34) and promoting the fungal growth. The present study’s findings align with these established mechanisms, as elevated ROS levels were detected in Zymosan A-stimulated iDC. Furthermore, augmented ROS-levels were identified in Dex-DC. In both Zymosan A-stimulated iDC and Dex-DC, the production of ROS could be abrogated by treatment with an anti-Dectin-1 antibody, thereby emphasizing the pivotal role of β-glucan-associated initial ROS production. It is acknowledged that ROS function as signals for NLRP3 inflammasome activation, though the precise mechanisms underlying this process remain to be fully elucidated. One potential mechanism involves the direct binding of mtDNA, released due to excessive generation of mtROS, to NLRP3, resulting in its subsequent activation (35). In order to assess whether the use of antioxidants in Dectin-1-activated dendritic cells (DC) leads to relevantly lower reactive oxygen species (ROS) levels, we tested the SOD-mimetic MnTBAP. This treatment has been shown to effectively reduce ROS levels in iDC; however, it does not approximate the reduced ROS levels observed in Dex-DC. Nevertheless, we can only indirectly conclude from our data that lower absolute ROS levels due to the use of MnTBAP result in lower amounts of mtROS. During systemic infections, excessive ROS production can lead to multi-organ system failures due to cellular dysfunctions (36). To overcome the complications of sepsis in steroid-treated patients with heightened vulnerability, the use of NLRP3-inhibitors as a therapeutic option might be considered. Furthermore, the use of antioxidants to protect the mitochondria could potentially reduce mitochondrial dysfunction and the release of mtDNA, which in turn could diminish NLRP3-activation and pyroptosis.

Up to date, the clinical application of NLRP3 inhibitors in patients treated with corticosteroids remains largely investigational, with several promising directions. So far no NLRP3 inhibitor is currently approved for human use, but a number of orally available inhibitors are entering and advancing in clinical trials, including compounds such as MCC950 (CRID3), dapansutrile, and NT-0796 (37). Early clinical trials suggest NLRP3 inhibitors are generally well-tolerated, with most adverse effects being mild (gastrointestinal symptoms, headaches), but long-term safety data are still pending (38). The use of NLRP3 Inhibition in fungal Infections might be benefical since targeting the NLRP3 inflammasome may dampen pathological inflammation seen during corticosteroid therapy, potentially reducing tissue damage and controlling excessive cytokine release. Though, a crucial venture might an increased risk of opportunistic or invasive fungal infections, especially in immunocompromised patients, since the NLRP3 inflammasome plays a key role in immune defense against pathogens (38, 39). Currently, specific clinical data on infection rates in patients on NLRP3 inhibitors are lacking, underscoring the need for ongoing surveillance and caution in future trials where fungal infection is a risk. Patients with excessive inflammation with a need of corticosteroid therapy (such as those with severe hematologic or autoimmune disease, acute lung injury, septic patients or some chronic inflammatory syndromes) or those at high risk of steroid-induced tissue damage but lower risk for severe infection (e.g., selected oncology or transplant populations, not acutely neutropenic) could benefit from NLRP3-targeted dampening of the innate immune response. This is exemplary shown for Patients with overactive or mutation-driven NLRP3 inflammasome activation (e.g., CAPS-Cryopyrin-Associated Periodic Syndromes), as demonstrated by functional variant screening with MCC950 (40). Clinical trial designs should provide essential insights on both the efficacy and infection risk profile - especially in settings where the delicate balance between immune suppression and protection is critical.

This study has some limitations such as *in vitro* models.The absence of *in vitro* analysis restricts the mechanistic insights that might be gained regarding direct pathogen-host interactions or drug responses under defined conditions and must be performed in future work. Furthermore, monocyte-derived DC from healthy donors instead of primary subjects (e.g., samples or cells directly derived from patients) were used as experimental model. Research employing primary tissues or patient-derived cells can offer translational relevance. However, in our setting, with acute fungal infections, it is almost impossible to obtain primary samples. The patients require emergency care, which means that the time window for scientific research is very narrow.

Taken together, these findings demonstrate an enhanced induction of IL-1ß and IL-18 in Dex-DC compared to iDC upon stimulation with β-glucans. In regard to the inhibitor experiments, the classical and alternative pathways of NLRP3 inflammasome activation are considered as potential sources for the amplified IL-1ß and IL-18 generation. Furthermore, an intensified fungal-associated inflammation was observed subsequent to steroid treatment, including dexamethasone. These effects can lead to higher mortality in patients suffering from local and systemic fungal infections with the need of steroid-treatment. Consequently, the use of NLRP3-inhibitors has the potential to ameliorate the clinical course in such patients and prevent fatal outcomes from uncontrolled sepsis.

## Data Availability

The original contributions presented in the study are included in the article/**Supplementary Material**, further inquiries can be directed to the corresponding author/s.

## Supplementary material

Supplementary Figure 1 Supernatants of iDC and Dex-DC with and without Dectin-activation via Curdlan-stimulation were analyzed for IL-10 secretion as described. Scatter bar plots show mean ± SEM with each dot representing the median of duplicates of three experiments. Mann-Whitney-U-test was applied for statistical analysis. **P* < 0.05

Supplementary Figure 2 Representative example of gating for ASC-Specks in human monocyte-dervied DCs.
