# Supplementary figures and images for: Dexamethasone induced Dectin-1 activation enhances NLRP3 inflammasome activation
Source: Front Immunol. 2025 Sep 12;16:1656288. doi: 10.3389/fimmu.2025.1656288 (PMC12465279; doi:10.3389/fimmu.2025.1656288)

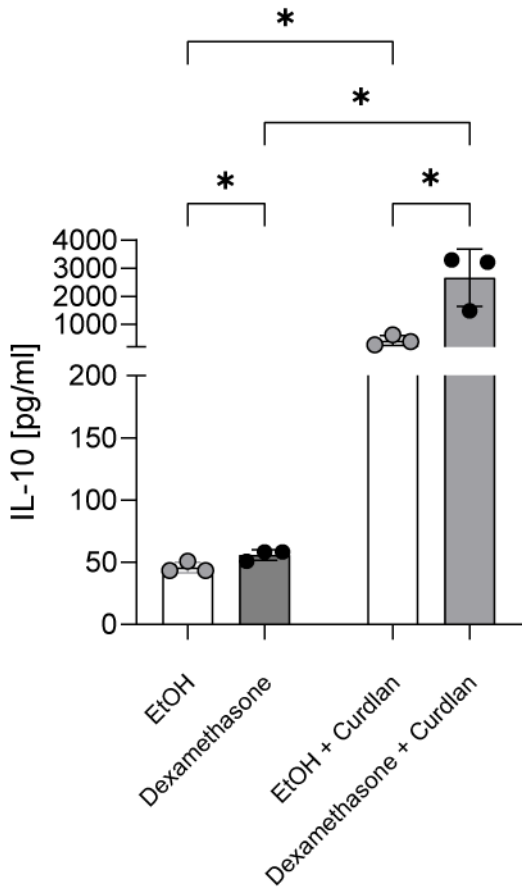

Supplement: Supplementary Figure 1 — Supernatants of iDC and Dex-DC with and without Dectin-activation via Curdlan-stimulation were analyzed for IL-10 secretion as described. Scatter bar plots show mean ± SEM with each dot representing the median of duplicates of three experiments. Mann-Whitney-U-test was applied for statistical analysis. *P < 0.05 [file DataSheet1.pdf]

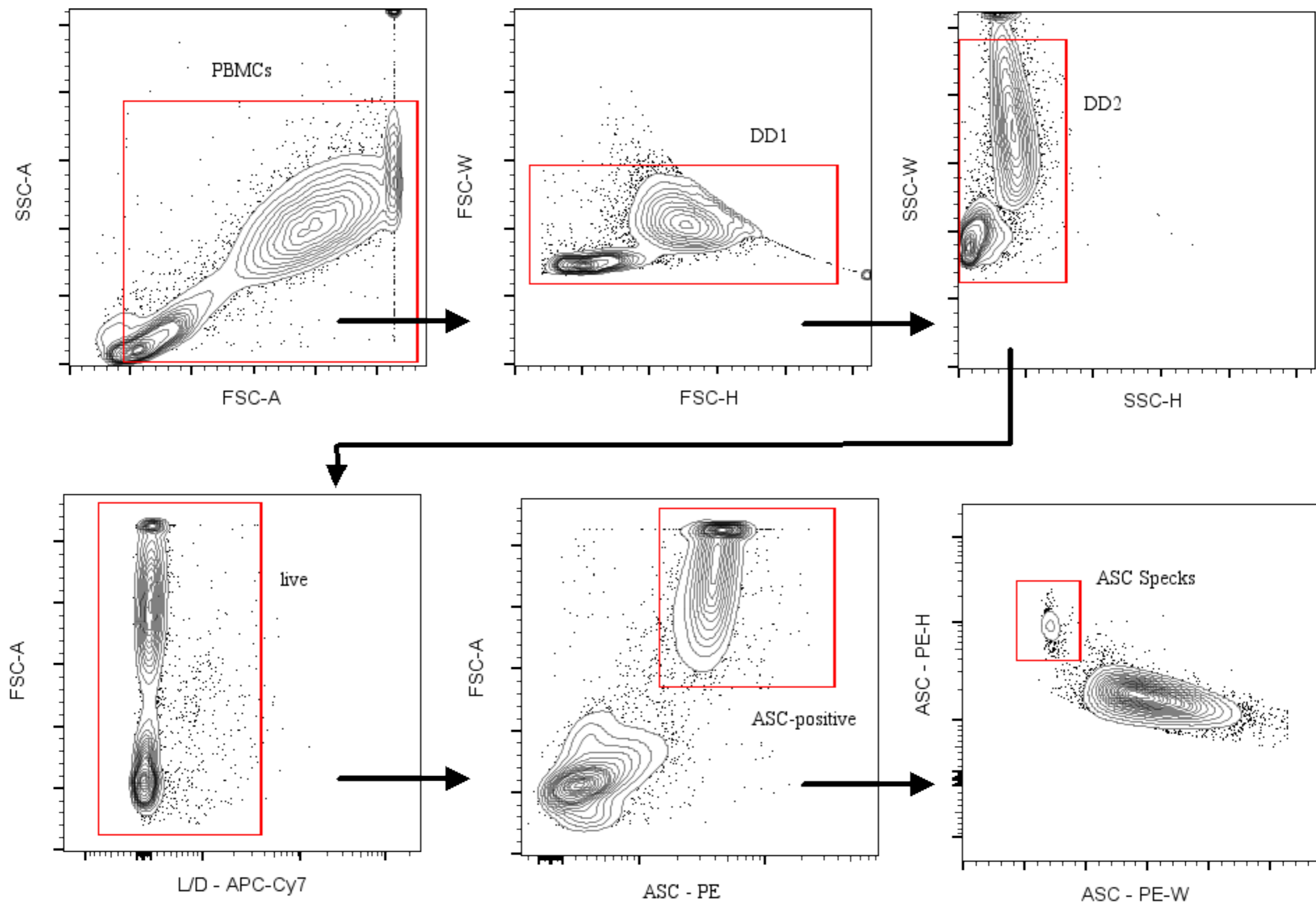

Supplement: Supplementary Figure 2 — Representative example of gating for ASC-Specks in human monocyte-dervied DCs. [file DataSheet2.pdf]
